# Regulation of hemolysin in uropathogenic *Escherichia coli* fine-tunes killing of human macrophages

**DOI:** 10.1080/21505594.2018.1465786

**Published:** 2018-05-30

**Authors:** Ambika M. V. Murthy, Minh-Duy Phan, Kate M. Peters, Nguyen Thi Khanh Nhu, Rodney A. Welch, Glen C. Ulett, Mark A. Schembri, Matthew J. Sweet

**Affiliations:** a Institute for Molecular Bioscience (IMB), IMB Centre for Inflammation and Disease Research, and the Australian Infectious Diseases Research Centre, The University of Queensland, QLD, Australia; b School of Chemistry and Molecular Biosciences, and the Australian Infectious Diseases Research Centre, The University of Queensland, QLD, Australia; c Department of Medical Microbiology and Immunology, University of Wisconsin School of Medicine and Public Health, Madison, WI, USA; d School of Medical Science, and Menzies Health Institute Queensland, Griffith University, QLD, Australia

**Keywords:** α-hemolysin, cell death, cof, gene regulation, inflammasome, macrophage, UPEC, urinary tract infection

## Abstract

Uropathogenic *E. coli* (UPEC) causes the majority of urinary tract infections (UTIs), which are a major global public health concern. UPEC uses numerous mechanisms to subvert the innate immune system, including targeting macrophage functions. We recently showed that some UPEC strains rapidly kill human macrophages via an NLRP3-independent pathway, and also trigger NLRP3-dependent IL-1β processing. In this study, we used random transposon mutagenesis in the reference strain CFT073 to identify UPEC genes that mediate human macrophage cell death. Our approach revealed that the hemolysin A (HlyA) toxin is essential for triggering both cell death and NLRP3 inflammasome-mediated IL-1β release in human macrophages. Random transposon mutagenesis also identified the *cof* gene, which encodes a poorly characterized phosphatase, as a novel hemolysin regulator; a CFT073 mutant deleted for the *cof* gene secreted significantly reduced levels of HlyA, had diminished hemolytic activity, and was impaired in its capacity to trigger human macrophage cell death and IL-1β release. Together, our findings reveal that Cof fine-tunes production of hemolysin, an important determinant of both UPEC-mediated inflammasome activation and human macrophage cell death.

## Introduction

Urinary tract infections (UTIs) are one of the most common bacterial infections, and can be associated with serious complications. Infection of the bladder (cystitis) can lead to kidney infection (pyelonephritis), associated renal damage and life-threatening sepsis [1,2]. The majority of acute and chronic UTIs are caused by uropathogenic *E. coli* (UPEC) [3]. Although antibiotics are routinely used to treat UTIs, the emergence of multidrug-resistant UPEC strains poses a significant challenge to effective treatment of such infections [4]. It is therefore crucial that we improve our current understanding of UPEC pathogenesis in order to develop novel strategies to prevent and/or treat these infections.

The innate immune system is composed of both soluble factors and cellular mediators that provide a coordinated response to enable UPEC detection and destruction. Although host-protective pro-inflammatory mediators such as IL-6 and IL-8 are released during active UTIs [5–7], several studies have also demonstrated that UPEC can actively suppress such responses [8–10]. The capacity of UPEC to downregulate a range of inflammation-related genes may contribute to the dampening of neutrophil recruitment to the site of infection [11]. For example, the UPEC periplasmic protein YbcL inhibits transepithelial neutrophil migration in *in vitro* and *in vivo* models of murine cystitis [12]. We recently demonstrated that CFT073, a virulent blood culture isolate recovered from a patient with pyelonephritis, triggers rapid cell death in both human and mouse macrophages [13]. Since macrophages play an essential role in the recruitment of host-protective neutrophils during experimental UTI [14], UPEC-mediated killing of macrophages may provide an additional mechanism by which this pathogen can evade neutrophil responses.

UPEC strains that kill macrophages also trigger activation of the nod-like-receptor pyrin domain-containing 3 (NLRP3) inflammasome [13]. Inflammasomes are large cytosolic multiprotein complexes that are activated by infection and/or cellular stress. They comprise a danger-sensor pattern recognition receptor, such as an NLR family member, as well as downstream signalling components. NLRP3, which is the best-characterized inflammasome-forming pattern recognition receptor, is activated by a wide array of host-derived and exogenous ligands, including microbial toxins [15]. Upon activation, NLRP3 oligomerizes in the presence of the adaptor protein apoptosis-associated speck-like protein containing a CARD (ASC) and the effector caspase-1, resulting in auto-proteolysis and activation of caspase-1. Activated caspase-1 cleaves the pro-inflammatory cytokines pro-IL-1β and pro-IL-18 enabling their release from cells, and also processes gasdermin D to enable pyroptotic cell death [16]. Several bacterial toxins such as nigericin (*Streptomyces hygroscopicus*), valinomycin (several *Streptomyces* species), maitotoxin (*Gambierdiscus toxicus*), aerolysin (*Aeromonas hydrophila*) and listeriolysin O (*Listeria monocytogenes*) activate the NLRP3 inflammasome [17]. Intriguingly, a UPEC factor that reduces inflammasome activation has also been described [18].

The success of UPEC as a pathogen partly reflects its versatility in deploying different virulence systems, for example toxins (e.g. hemolysin, cytotoxic necrotizing factor, autotransporters), adherence factors (e.g. fimbriae) and nutrient acquisition systems (e.g. iron uptake systems) [19–21]. Of the known UPEC toxins, hemolysin (HlyA), a pore-forming toxin that is present in approximately 50% of UPEC strains [22], plays a significant role in pathogenesis [23]. Full-length HlyA, which is 110 kDa in size, is secreted by the gene products of the *hlyCABD* operon, as well as TolC that is not genetically linked to this operon [24]. The immature pro-HlyA, which is encoded by *hlyA*, is post-translationally modified by HlyC-mediated acylation at Lys564 and Lys690, resulting in the mature form of HlyA [25]. This processing of HlyA is required for its cytotoxic and lytic activities [26]. HlyB and HlyD, along with TolC, form a type I secretion complex that aids in the secretion of HlyA across the inner and outer membranes [27]. Binding of Ca^2+^ ions to the C-terminal portion of HlyA is also necessary for its cytotoxic activity [28].

Our previous studies showed that CFT073 initiated rapid cell death in an NLRP3-dependent manner in mouse macrophages, whereas in human macrophages, cell death still proceeded when NLRP3 was pharmacologically blocked. A CFT073 *hlyA* mutant was defective in initiating cell death and IL-1β maturation and release in mouse macrophages, whereas this mutation only partially reduced the amount of UPEC-triggered cell death in human macrophages [13]. In this study, we generated a random transposon mutant library and used this to identify UPEC genes involved in CFT073-triggered cell death in human macrophages. Our studies reveal a dominant role for HlyA in initiating this response, and identify the Cof phosphatase as a previously unknown regulator of HlyA production, and thus human macrophage cell death.

## Results

### Identification of CFT073 genes involved in initiation of human macrophage cell death

**Figure 1.**
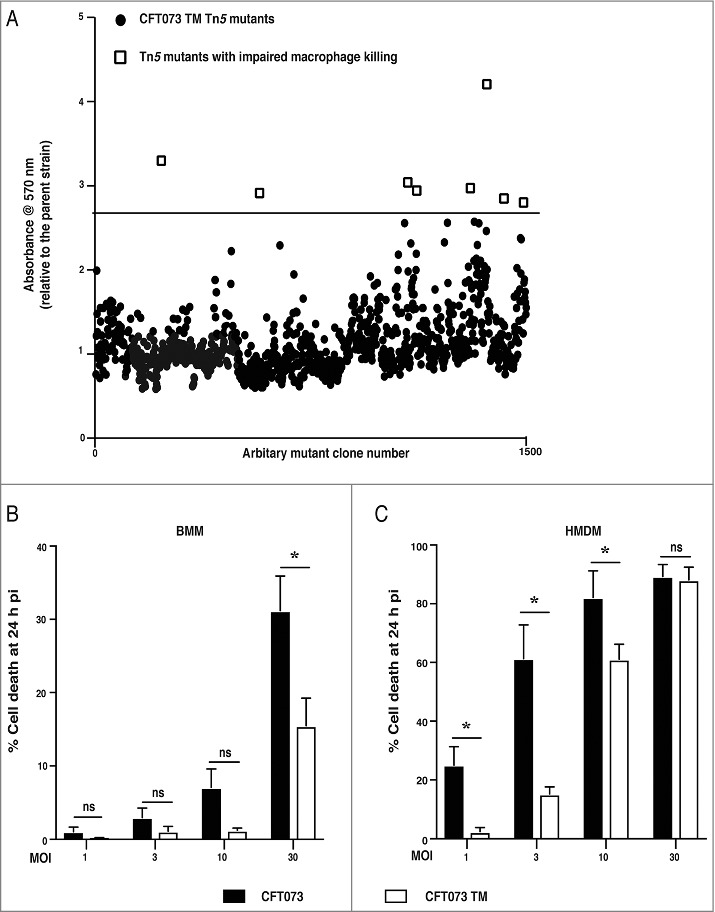
Screening of a transposon mutant library for mutants with a reduced capacity to kill human macrophages. (A) HMDM were treated with 50 μL of CFT073 TM mutant cultures (grown overnight) from individual Tn*5* mutant clones, as well as the parental CFT073 TM strain. MTT assays, as a correlate of cell viability, were performed at 4 h post infection (pi), and a dot plot of HMDM viability after treatment with individual CFT073 TM Tn*5* mutants is shown. Each data point represents A_570_ nm, relative to the parental CFT073 TM strain that was included in each plate, in order to correct for plate-to-plate variation across the screen. The eight mutants above the arbitrary threshold (2.75 fold or greater MTT value than that of the parental CFT073 TM strain for that plate) are displayed as open boxes. (B-C) BMM (B) or HMDM (C) were infected with either wild type CFT073 (filled bars) or the CFT073 TM (unfilled bars) over an MOI range (1, 3, 10 and 30), as indicated. Cell death was analyzed at 24 h pi by LDH release assays. Data (mean+sem; n = 5) are combined from five independent experiments, and each experiment was performed in experimental triplicate. Statistical significance was determined by a two-tailed t-test (*p < 0.05). ns = not significantly different.

We used an unbiased approach of random mutagenesis to identify candidate UPEC virulence factor(s), and their regulators, that mediate human macrophage cell death. We first generated a random transposon mutant library in the CFT073 TM strain [13], which harbours deletions in the genes encoding the secreted autotransporter toxin (sat) and the vacuolating autotransporter protein (vat), as well as an insertion of the kanamycin resistance gene into the 5′ region of *hlyA*, disrupting production of the full-length protein. We previously found that, despite these mutations, this strain still kills primary human macrophages [13]. We then used a semi high-throughput screen, based on the 3-(4,5-dimethylthiazol-2-yl)-2,5-diphenyltetrazolium bromide (MTT) assay [29], which measures NADPH-dependent cellular oxidoreductase enzyme activity. This assay is widely used as an indirect measure of cell viability, and thus served as a primary screen to identify candidate mutants defective in their ability to kill primary human monocyte-derived macrophages (HMDM). EC958, a reference ST131 strain isolated from the urine of a UTI patient, does not kill HMDM [13] and was thus used as a negative control in these experiments. Prior to infection, the optical density at 600 nm (OD_600_) was also recorded to ensure that the mutants had a similar growth rate to the parent CFT073 TM strain and EC958. Using this approach, we identified eight mutants (out of ∼1500 screened) that displayed a reduced capacity to kill HMDM, as determined by a ≥ 2.75-fold increase in the MTT reading for that mutant compared to the parental strain (Figure 1A).

Of the mutants identified, three (CFT073 TM_01, CFT073 TM_05 and CFT073 TM_08) showed a clear phenotype in subsequent lactate dehydrogenase (LDH) release assays (a more direct measure of cell death), by comparison to the CFT073 TM parent strain, as well as two other randomly-selected transposon mutant controls (NC 1, NC 2) that behaved in a similar fashion to the parent strain (Supplementary Figure 1A and B). In contrast, more subtle effects were apparent for the other mutants, with reduced killing only observed at a multiplicity of infection (MOI) of 1 at 24 h post-infection (with the exception of TM_04 that showed no phenotype under any conditions). To identify the gene(s) in which the transposon was inserted, an arbitrary PCR was performed, after which the amplicons were sequenced to identify the targeted gene(s) in CFT073 (Supplementary Table 1). The CFT073 TM_01 and CFT073 TM_05 mutants contained Tn*5* insertions in *hlyA*, whereas CFT073 TM_08 contained a Tn*5* insertion in the *cof* gene, which encodes a protein with phosphatase activity [30]. We confirmed that the TM_01 and TM_05 mutants were deficient in HlyA expression and activity by immunoblotting (Supplementary Figure 2A) and by hemolytic activity assays on sheep red blood cells (Supplementary Figure 2B). Interestingly, the TM_08 mutant had diminished HlyA expression (Supplementary Figure 2, A and C) and hemolytic activity (Supplementary Figure 2B), implying that Cof may be a regulator of this toxin (see ahead).

The identification of *hlyA* in our screen was surprising, since we performed our transposon screen on CFT073 TM, which contains an insertion in this gene. We previously found that this mutation substantially reduced UPEC-mediated killing of primary mouse bone marrow-derived macrophages (BMM) [13]. However, the mutation in this strain does not completely delete *hlyA*, rather the gene was disrupted by insertion of a kanamycin resistance cassette 72 nucleotides downstream of the start codon. Shorter forms of HlyA are known to be generated from internal translation start sites [31], and thus it was conceivable that the CFT073 TM strain generates partially active truncated forms of HlyA through the use of an alternative translation start site(s) downstream of the kanamycin cassette. We therefore reexamined the capacity of CFT073 TM to kill BMM over an MOI range, finding that it did cause substantial UPEC-induced cell death at the highest MOI examined (MOI 30) (Figure 1B). Conversely, by comparison to wild type CFT073, killing of HMDM by this strain was substantially reduced at lower MOIs (MOI 1, 3) (Figure 1C). Collectively, these data suggest that disruption of *hlyA* in CFT073 TM impairs, but does not ablate, HlyA-mediated killing of human and mouse macrophages. The findings also support the view that HMDM are more sensitive than BMM to this toxin, and the full-length HlyA is required for optimal killing of macrophages.

### Hemolysin is required for CFT073-mediated cell death and IL-1β release in human macrophages

**Figure 2.**
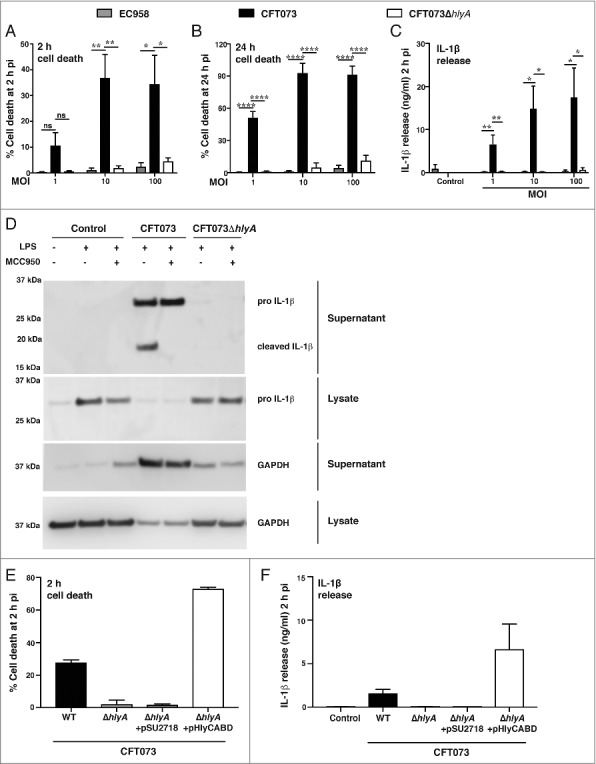
Hemolysin is required for CFT073-mediated cell death and IL-1β release in human macrophages. HMDM were left untreated (A-B) or primed with LPS (100 ng/ml) for 4 h (C), prior to infection with the indicated UPEC strains over an MOI range. (A-B) Culture supernatants were collected at the indicated time points, and analyzed for LDH release as an indicator of cell death. (C) Culture supernatants (2 h pi) were analyzed for IL-1β release by ELISA. Data (mean+sem; n = 4) are combined from four independent experiments, and each experiment was performed in experimental triplicate. Statistical significance was determined by one-way ANOVA, followed by a Dunnett's test for multiple comparison (*p < 0.05, **p<0.01, ****p < 0.0001). ns = not significantly different. (D) HMDM were left untreated or were primed with LPS (100 ng/ml) for 4 h, then pre-treated with the NLRP3-specific inhibitor, MCC950 for 1 h prior to infection with the indicated UPEC strains (MOI 10). Cell lysates and supernatants were collected at 2 h pi, and were analyzed by western blot for pro- and cleaved IL-1β. Glyceraldehyde 3-phosphate dehydrogenase (GAPDH) was used as a loading control and as an additional read-out for cell death (release into cell culture supernatants). Similar results were apparent in two independent experiments. (E-F) HMDM were left untreated (E) or primed with LPS (100 ng/ml) for 4 h (F), prior to infection with the indicated UPEC strains (WT denotes wild type, pSU2718 is the empty vector control, pHlyCABD is a plasmid expressing the entire hemolysin operon) at MOI 10. (E) Culture supernatants were collected at 2 h pi and analyzed for LDH release. (F) Culture supernatants (2 h pi) were analyzed for IL-1β release by ELISA. Data is from single experiment performed in experimental triplicate (mean+range), and similar observations were made in three independent experiments.

Given the findings from our transposon mutagenesis screen, we next deleted *hlyA* from CFT073 by gene displacement using λ-Red homologous recombination. The resultant complete deletion mutant (CFT073Δ*hlyA*) failed to initiate HMDM cell death over an MOI range at 2 h and 24 h post-infection, with any residual effect being similar to that observed with the *hlyA*^−ve^ ST131 strain EC958 (Figure 2A and B). Previous studies from our laboratory have shown that UPEC-triggered cell death correlates with inflammasome activation and release of mature IL-1β [13]. Accordingly, UPEC-triggered release of IL-1β from LPS-primed HMDM was abrogated when using CFT073Δ*hlyA* (Figure 2C). To examine the role of the NLRP3 inflammasome, we assessed the effect of the NLRP3-specific inhibitor MCC950 [32] on UPEC-triggered IL-1β release from LPS-primed HMDM by immunoblotting. As expected, cleaved (bioactive) IL-1β was released from HMDM infected with wild type CFT073, with this effect being antagonized by MCC950 (Figure 2D). In contrast, MCC950 did not inhibit release of unprocessed pro-IL-1β, which likely occurs as a result of cell death (as evidenced by GAPDH release into culture supernatants, Figure 2D). Notably, CFT073Δ*hlyA* failed to elicit either release of processed IL-1β or cell death (release of pro-IL-1β and GAPDH) (Figure 2D). Collectively, these results demonstrate that HlyA is the primary mediator of both NLRP3-independent cell death and NLRP3-dependent IL-1β release in HMDM. To further validate the role of HlyA in these responses, we complemented CFT073Δ*hlyA* with a plasmid expressing the entire hemolysin operon (pHlyCABD). Complementation of CFT073Δ*hlyA* with this plasmid restored both cell death and IL-1β release, whereas the empty vector (pSU2718) had no effect (Figure 2E–F).

### Cof is essential for maximal CFT073-triggered HMDM cell death and IL-1β release

**Figure 3.**
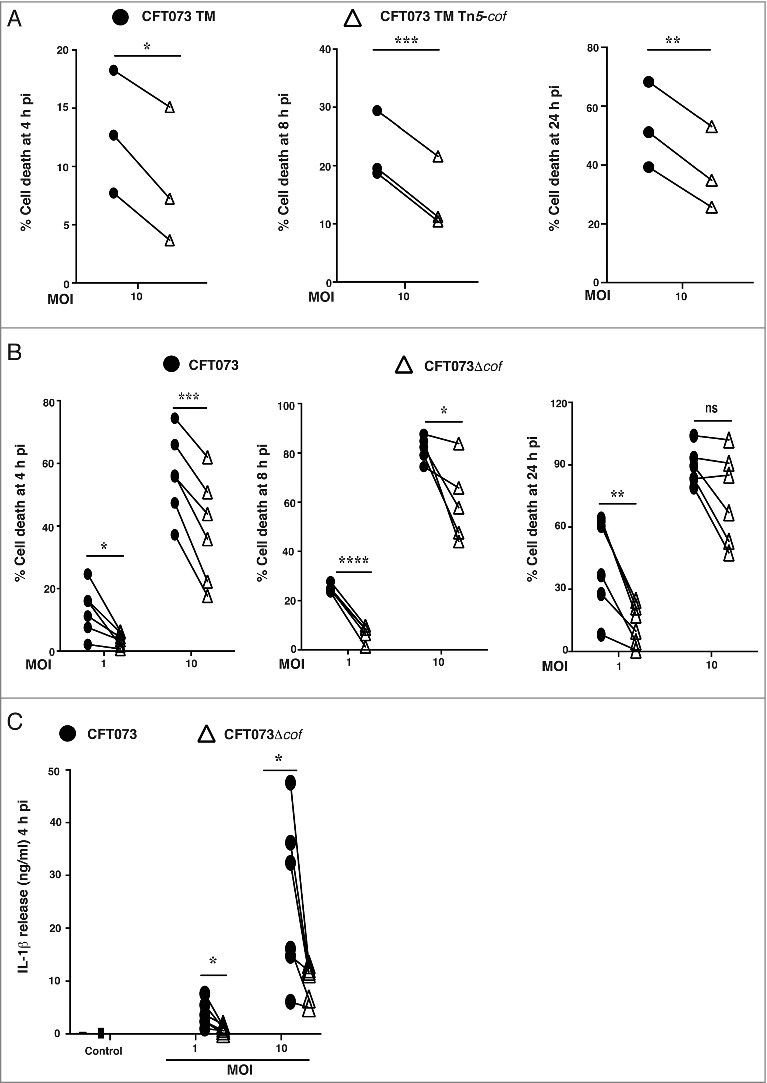
*cof* is required for maximal CFT073-mediated human macrophage cell death. (A) HMDM were infected with CFT073 TM or the CFT073 TM Tn*5*-*cof* mutant at MOI 10. Cell death was measured at 4, 8 or 24 h pi using LDH release assays. Data (mean + sem; n = 3) were combined from three independent experiments performed in experimental triplicate (each experiment using a different donor). Statistical significance was determined by a two-tailed t-test (*p < 0.05, **p < 0.01, ***p < 0.001). (B) HMDM were infected with wild type CFT073 or the CFT073Δcof over the indicated MOI range. Cell death was quantified by LDH release assays, using supernatants collected at 4, 8 or 24 h pi. Data (mean+sem; n = 6) are combined from six independent experiments performed in experimental triplicate (each experiment using a different donor). Statistical significance was determined by a paired two-tailed t-test (*p < 0.05, **p<0.01, ***p < 0.001, ****p < 0.0001). ns = not significantly different. (C) HMDM were primed with LPS (100 ng/ml) for 4 h prior to infection with the indicated strains. Culture supernatants were analyzed for IL-1β release at 4 h pi. Data (mean + sem; n = 6) are combined from six independent experiments performed in experimental triplicate (each experiment using a different donor). Statistical significance was determined by a two-tailed t-test (*p < 0.05). Connecting lines represent the results of a single experiment using one donor (for CFT073 TM vs CFT073 TM Tn*5*-*cof* or CFT073 vs CFT073Δ*cof*).

We next focused on the poorly characterized *cof* gene [30], since it was identified as a potential UPEC gene involved in initiating HMDM cell death (Supplementary Table 1). Compared to the parental CFT073 TM strain, CFT073 TM Tn*5*-*cof*-mediated killing of HMDM was reduced over a time course (4 – 24 h) (Figure 3A). We then generated an independent *cof* deletion mutant in wild type CFT073 (referred to as CFT073Δ*cof)*, and observed a similarly reduced capacity to initiate HMDM cell death at multiple time points, with the effect being most pronounced at the lowest MOI (Figure 3B). As expected, CFT073Δ*cof*-mediated IL-1β release from LPS-primed HMDM was also diminished compared to wild type CFT073 (Figure 3C). Collectively, these data indicate that *cof* is required for maximal CFT073-triggered cell death and IL-1β release in HMDM.

### Cof regulates levels of secreted hemolysin

**Figure 4.**
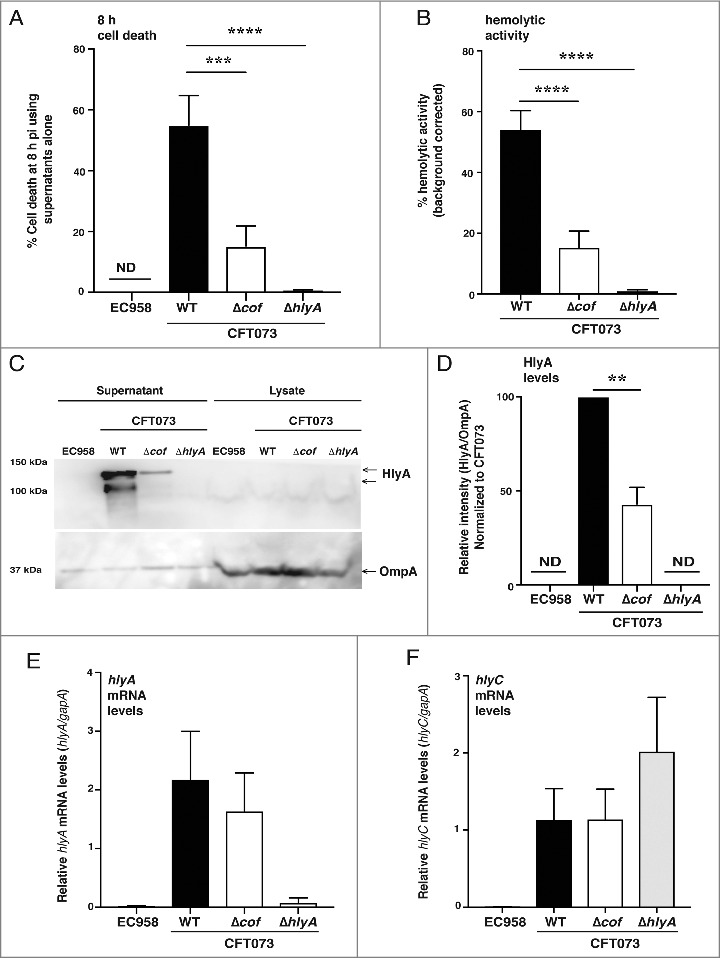
Cof regulates levels of secreted hemolysin. (A) HMDM were treated with the supernatants from the indicated bacterial strains (WT denotes wild type). Culture supernatants were analyzed for cell death at 8 h pi, using LDH release assays. Data (mean+sem, n = 3) are combined from three independent experiments performed in experimental triplicate (each experiment using a different donor). Statistical significance was determined by one-way ANOVA, followed by Dunnett's multiple comparison test (***p < 0.001, ****p < 0.0001). (B) Overnight cultures of the indicated UPEC strains (WT denotes wild type) were incubated for 4 h with 5% sheep blood, after which hemolytic activity was measured. The *hlyA*^−ve^ strain EC958 was used for background correction in these experiments, and data (mean+sem; n = 3) are combined from three independent experiments performed in experimental triplicate. Statistical significance was determined by one-way ANOVA, followed by Dunnett's multiple comparison test (****p < 0.0001). (C) Supernatants from the indicated bacterial strains (grown overnight) were concentrated (WT denotes wild type) and lysates from matched cell pellets were generated. The samples were analyzed for hemolysin expression by western blotting using antibodies targeting HlyA (the full-length HlyA migrated at ∼120 kDa, in contrast to its predicted size of ∼110 kDa). OmpA expression was used as a loading control for the cell lysates. Similar results were obtained in five independent experiments. (D) Levels of HlyA in supernatants, relative to OmpA levels in lysates from matched cell pellets for each strain, were determined by immunoblotting (using ImageJ/Image analyzer (Amersham 600)). For each experiment, the HlyA levels in wild type CFT073 were set at 100%. Data (mean+sem; n = 5) are combined from five independent experiments. Statistical significance was determined by one-way ANOVA, followed by Dunnett's multiple comparison test (**p < 0.01). (E-F) Levels of *hlyA* (E) and *hlyC* (F) mRNA, relate to *gapA*, in the indicated UPEC strains (WT denotes wild type) were determined by qRT-PCR. Data (mean+sem; n = 4) are combined from four independent experiments. ND = not detected.

Given our observation that the CFT073 TM_08 *cof* mutant had diminished HlyA expression and hemolytic activity (Supplementary Figure 2A–C), we postulated that Cof likely regulates the expression, secretion or stability of this toxin. As hemolysin is released into bacterial culture supernatants [33], we treated HMDM with supernatants that were prepared from liquid cultures of UPEC and monitored the cell death response. Supernatants from *CFT073Δcof* showed a significantly reduced capacity to kill HMDM compared to supernatants prepared from wild type CFT073 (Figure 4A). In addition, supernatants from control bacteria that do not produce HlyA (EC958 and CFT073Δ*hlyA*) failed to kill macrophages. As observed with the CFT073 TM_08 *cof* mutant versus the CFT073 TM control (Supplementary Figure 2B), we found that CFT073Δ*cof* showed a significant reduction in hemolytic activity compared to wild type CFT073 (Figure 4B). To determine if Cof promotes cell death by regulating HlyA, we quantified levels of HlyA in the supernatants of wild type CFT073 and *CFT073Δcof* by immunoblotting. In these experiments, we observed a significant reduction in full-length HlyA secreted by CFT073Δ*cof* compared to wild type CFT073. Lower molecular weight forms of HlyA, which likely reflect truncated products generated by internal translation start sites [31] and/or degradation products, were also significantly reduced in CFT073Δ*cof* (Figure 4C & D). To determine whether this reduction was due to impaired secretion, levels of HlyA in cell lysates were also assessed. This analysis revealed no build-up of HlyA in CFT073Δ*cof* cell lysates compared to lysates prepared from wild type CFT073 (Figure 4C). To determine whether the defect in levels of HlyA may be linked to *hlyA* transcription, we next analyzed mRNA levels by quantitative RT-PCR (qRT-PCR). We found no difference in the levels of *hlyA* mRNA in wild type CFT073 versus CFT073Δ*cof* (Figure 4E). As HlyC is required for the conversion of pro-HlyA to active HlyA, we also measured the mRNA levels of *hlyC*, again observing no difference in the transcript levels of this gene (Figure 4F). Taken together, these results indicate that Cof controls the levels of secreted HlyA in CFT073, with translational or post-translational regulation likely to account for these effects.

## Discussion

Some bacterial pathogens that occupy an intracellular niche, for example *Salmonella enterica* serovar Typhimurium, engage inflammasome pathways, thus resulting in the initiation of pyroptotic cell death. This inflammatory form of cell death likely serves to expose intracellular bacteria to extracellular host defence mechanisms, such as neutrophil-mediated killing [34]. Although some strains of UPEC can reside in epithelial cells [35] and macrophages [36], others trigger inflammasome activation or rapid cell death [13]. Whether UPEC-triggered macrophage cell death is beneficial to the host or to the pathogen is unclear at this stage, and knowledge of both host and pathogen factors that are involved in this response are required to address this question. We previously showed that CFT073 activates the NLRP3 inflammasome, leading to IL-1β maturation, in both human and mouse macrophages [13]. However, cell death still proceeded in the absence of NLRP3 in human macrophages, whereas this response was strictly NLRP3-dependent in mouse macrophages. We also observed that CFT073 TM, used initially in these studies, was more profoundly compromised for killing of primary mouse macrophages than human macrophages [13]. Consequently, we had proposed the existence of alternative UPEC toxins triggering macrophage cell death, but now find that the disrupted HlyA in CFT073 TM retains partial activity (Figure 1B–C, Supplementary Figure 2B). This suggests that the full-length toxin is particularly important for initiating macrophage cell death. We conclude that the differences between human and mouse with respect to HlyA involvement in macrophage killing likely reflect the heightened sensitivity of human macrophages to HlyA-mediated cell death.

Many bacterial factors, including toxins, LPS and DNA are known to activate inflammasomes in macrophages. In this study, we show that HlyA toxin is essential for triggering cell death and IL-1β release in human macrophages. The prevalence of UPEC strains that carry this toxin increases with the severity of infection, with up to 78% of pyelonephritis-causing UPEC strains encoding HlyA [37–39]. At high concentrations, HlyA forms lytic pores in cell membranes [40]. However, at sublytic concentrations, which are most likely to be relevant to the *in vivo* scenario during an active UTI, HlyA degrades paxillin and other host proteins that are involved in cell-cell and cell-matrix interactions, a mechanism known to cause exfoliation [8]. Exfoliation has been observed in human bladder epithelial cells in the three dimensional organoid model *in vitro [*41], as well as *in vivo* in a mouse UTI model [42]. HlyA is also known to dephosphorylate the Akt protein in bladder epithelial cells, which may decrease pro-inflammatory responses and increase pro-apoptotic events, leading to exfoliation [43,44]. Another study in the UTI89 strain demonstrated that HlyA triggered uroepithelial cell death and IL-1α release via caspase-4 activation, as well as NLRP3-mediated IL-1β release and cell death [45]. HlyA has also been reported to initiate cell death in monocytes, neutrophils and T-lymphocytes [46–49]. Such effects may contribute to host colonization through several means. Firstly, epithelial cell death could expose underlying tissue to bacteria. Secondly, rapid killing of macrophages by UPEC may limit TNF-α production [13], which is a required component of the complex cellular network that orchestrates neutrophil recruitment during experimental UTI [14]. Conversely, it is possible that HlyA-triggered cell death may play a host-protective role in some settings. For example, it may expose intracellular bacteria to immune responses in the extracellular environment, and inflammasome-mediated IL-1β maturation and release would also be predicted to contribute to the recruitment of host-protective neutrophils [50].

Part of our strategy for undertaking this study was to identify novel regulators of toxin-induced killing of macrophages. In so doing, we identified *cof*, and showed that it has a hitherto unrecognized role as a regulator of hemolysin, and thus, UPEC-mediating killing of macrophages. Deletion of *cof* in CFT073 reduced levels of secreted hemolysin, but had no effect on *hlyA* mRNA expression (Figure 4E). This suggests that this phosphatase is likely to act directly or indirectly on the HlyA protein, presumably at a translational and/or post-translational level. The *hlyCABD* operon is transcribed into a single mRNA [51], and no intracellular accumulation of HlyA protein in CFT073Δ*cof* was detected (Figure 4C). Thus, it seems likely that Cof may function in this pathway by either promoting HlyA translation or affecting HlyA turnover, for example by directly or indirectly stabilizing this toxin before it is secreted. Unlike our findings with CFT073Δ*hlyA* (Figure 2E–F), complementation of CFT073Δ*cof* with a Cof expression plasmid did not rescue the cell death and IL-1β release phenotypes (data not shown). Nonetheless, we observed similar phenotypes (i.e. reduced levels of secreted HlyA, hemolytic activity and killing of human macrophages) with two independent genetic approaches (Tn*5* mutant, targeted deletion). It therefore seems likely that optimal levels of Cof are required in order for it to control levels of secreted HlyA. This is perhaps not surprising, given that *cof* encodes a phosphatase and its expression may need to be tightly controlled to enable proper functioning and avoid pleiotropic effects in the cell. Cof belongs to the haloacid dehydrogenase (HAD) superfamily of phosphatases. On the basis of sequence comparisons of *E. coli* Cof with other members of this family, Cof was originally proposed to act as a phosphatase on phosphorylated sugars, which are involved in a range of cellular processes from amino acid biosynthesis to inactivation of toxins [52]. Cof was subsequently demonstrated to catalyse the conversion of 4-amino-2-methyl-5-hydroxymethylpyrimidine pyrophosphate to 4-amino-2-methyl-5-hydroxymethylpyrimidine phosphate [53]. As such, Cof negatively regulates the thiamine pyrophosphate biosynthetic pathway, and also confers resistance to certain antibiotics such as bacimethrin that are converted into toxic products by thiamine biosynthetic enzymes. Thus, it is possible that Cof plays dual roles in pathogenesis in UPEC, both by contributing to optimal HlyA production as well as mediating resistance to antibiotics that exert their toxicity via this mechanism. Cof is also present in other pathogenic Gram-negative bacteria including *Salmonella enterica*, *Klebsiella pneumoniae, Shigella* and *Citrobacter* species, and is highly conserved (∼80% sequence identity) across these species.

Perhaps not surprisingly, given that it regulates thiamine pyrophosphate biosynthesis, Cof is present in both HlyA^+ve^ and HlyA^−ve^ UPEC strains. This is also true for genes encoding other known hemolysin regulators, for example *rfaH* and fumarate and nitrate reductase (*fnr*). RfaH positively regulates *hlyA* by promoting efficient elongation of the *hlyCABD* transcript [51,54–56], but is also required for the expression of other virulence determinants including LPS and capsule formation [57]. Similarly, FNR is required for optimal expression of several virulence factors in CFT073, including type I fimbrae, P fimbrae and hemolysin [58]. Consequently, a CFT073 *fnr* mutant was defective in adherence and invasion into bladder and kidney epithelial cells, and displayed attenuated virulence in a mouse model of UTI [58]. Interestingly, FNR was shown to promote hemolysin expression only under anaerobic conditions, which likely enables inducible expression of this toxin in specific circumstances *in vivo*. Whether Cof, like RfaH and FNR, also regulates other UPEC virulence factors remains to be determined.

In conclusion, this study demonstrates that HlyA plays an essential role in CFT073-mediated cell death and IL-1β release in human macrophages. Cof, a poorly characterized phosphatase that controls the thiamine pyrophosphate biosynthesis pathway, fine-tunes levels of secreted hemolysin, and thus inflammasome responses and human macrophage cell death. Given the essential role of thiamine pyrophosphate in numerous metabolic pathways, we speculate that Cof-mediated control of hemolysin expression may provide a means of enhancing expression of this toxin to counteract host-directed metabolic stress. Further characterization of Cof is now required to determine the precise mechanisms by which it regulates HlyA, and potentially other virulence factors.

## Materials and methods

### Bacterial strains

The UPEC strains CFT073 [59] and CFT073 TM [13] have previously been described. CFT073 TM is deleted for *sat* and *vat*, and contains an insertion of the kanamycin resistance gene at nucleotide position 72 relative to the *hlyA* start codon. CFT073Δ*hlyA* and CFT073Δ*cof*, which contain complete deletions of the indicated gene, were generated using the λ-Red mediated homologous recombination employing a 3-way PCR strategy to generate 500 bp flanking regions at each end of the two genes as previously described [60–62]. The primers used for amplification of the kanamycin resistance gene and subsequent replacement of *hlyA* or *cof* in the CFT073 genome were as follows: *kan*_Fwd (5′-aggacgcccgccataaactg-3′); *kan*_Rev (5′-ggtttaacggttgtggacaac-3′); *hlyA*_Fwd upstream (5′-ctgcaatacgggctaaccaa-3′); *hlyA*_Rev upstream (5′-ggaataggaactaaggaggaggattgctttgcagactgtagtg-3′); *hlyA*_Fwd downstream (5′-cctacacaatcgctcaagactccggtaatgccagtgatt-3′); *hlyA*_Rev downstream (5′-gacgtccatcctctctccag-3′); *cof*_Fwd upstream (5′-gccattgtccgcccataaacgg-3′); *cof*_Rev upstream (5′-ggaataggaactaaggaggaggttcaggcatcaataaagtgccatcc-3′); *cof*_Fwd do-wnstream (5′-cctacacaatcgctcaagacgccgaaatcaggctg-3′); *cof_*Rev downstream (5′-gggcgggatctccacaagcatatgg-3′); *hlyA*_Fwd_screen (5′-gccagttccccattacacag-3′); *hlyA*_Rev_screen (5′-tgcccctgatataacgcctc-3′); *cof_*Fwd screen (5′-gcccggttgtagcggttcgagcg-3′); *cof_*Rev screen (5′-ggcatcagactgcttttccc-3′). CFT073Δ*hlyA* and CFT073Δ*cof* were confirmed by PCR using the primers *hlyA*_Fwd_screen/*hlyA*_Rev_screen and *cof_*Fwd screen/*cof_*Rev screen, respectively, and by DNA sequencing. For infection assays, all bacterial strains were grown in Luria-Bertani (LB) broth at 37°C overnight (with shaking at 200 rpm); the growth rate of all mutants was indistinguishable from wild type CFT073. For hemolysin complementation studies, the entire *hlyA* operon (*hlyCABD*) was PCR amplified (primers used were 5′-gcggagctcttaaagaggagaaaggtaccgcttggtttgcttttttttacctgc-3′ and 5′- gggatctagattaacgctcatgtaaactttctgttac-3′) from CFT073 genomic DNA, then cloned into the pSU2718 vector (the plasmid is referred to as pHlyCABD). The 5′ and 3′ ends of the *hlyA* operon sequence within this construct were confirmed by sequencing. CFT073Δ*hlyA* was transformed with either pSU2718 (empty vector) or pHlyCABD for functional studies.

### Generation of the transposon mutant library

A mini Tn*5* consisting of the chloramphenicol (Cm) gene cassette flanked by Tn*5* mosaic ends was generated by PCR using the primers 5′-ctgtctcttatacacatctcacgtcttgagcgattgtgtagg and 5′-ctgctcttatacacatctgacatgggaattaggccatggtcc. Transposomes were created using the amplification product, which was then phosphorylated using T4 polynucleotide kinase and mixed with EZ-Tn*5* transposase (Epicentre Biotechnologies), as per the manufacturer's instructions. Electrocompetent CFT073 TM cells were prepared as described earlier [63] and were electroporated with transposomes, further recovered with super optimal broth with catabolite repression media for 2 h at 37°C and were plated on LB plates containing chloramphenicol. From the colonies obtained, ∼1500 were screened for the human macrophage cell death response. The Tn*5* insertion site in mutants that exhibited an impaired macrophage cell death phenotype was identified by arbitrary PCR using the following primers: 5′-cgtaattccggatgagcatt*,* 5′-ggccacgcgtcgactagtac(n)^10^gatat, 5′*-*ggccacgcgtcgactagtac(n)^10^acgcc, 5′-ggccacgcgtcgacttagtac and 5′-ggccacgcgtcgactagtac.

### Mammalian cell culture

All experiments using primary human and mouse cells were approved by the relevant University of Queensland Ethics Committees. Human monocytes were isolated from the buffy coats of healthy donors (kindly provided by the Australian Red Cross) using CD14^+^ beads and MACS, after which they were differentiated into HMDM, as previously described [64]. Each experiment used cells from different donors. Murine BMM were generated by differentiating bone marrow cells from C57BL/6 mice with recombinant colony stimulating factor-1 (10,000 U/mL, Chiron) for 7 days. Mammalian cells were seeded at a density of 80,000/0.2 mL in 96 well plates (Nunc), and 4 h prior to infection, medium was replaced by fresh medium (RPMI 1640 supplemented with 2 mM glutamine and 10% fetal bovine serum) (Life Technologies). For IL-1β ELISAs, cells were primed with 100 ng/mL ultrapure LPS from *Salmonella minnesota* R595 (Invivogen) for 4 h, prior to infecting them with the indicated CFT073 strains to initiate inflammasome activation.

### Infection assays

Overnight cultures of UPEC strains were pelleted, washed with phosphate buffer saline and resuspended at the same optical density (OD_600_ = 0.45). The MOI used in experiments was confirmed by counting colony-forming units after serial dilution. At 1 h post-infection, 200 μg/mL gentamicin (Life Technologies) was added to prevent extracellular bacterial growth. At 2 h post-infection, cell culture supernatants were replaced with fresh medium containing 20 μg/mL gentamicin. For treatment of macrophages with UPEC culture supernatants alone, overnight cultures of UPEC strains were adjusted to an OD_600_ of 1.0 before aliquots were taken. In these experiments, bacterial cultures were pelleted, and supernatants were passed through 0.22 μm filters (polyethersulfone membrane, Millipore) to remove intact bacterial cells and associated debris. 50 μL of bacterial supernatants were added to the HMDM monolayer on 96 well plate. Control wells received 50 μL of LB media alone.

### Semi-high throughput protocol for transposon mutant screening

In order to overcome the variability associated with different donors, monocytes from 10 independent donors were isolated, pooled and then frozen in aliquots. One week prior to commencing the screening, a batch of monocytes was recovered and differentiated with colony stimulating factor-1 (10,000 units/mL, Chiron). For transposon mutant screening, single mutant colonies were grown overnight at 37°C on 96 well plate. As part of the screen, each plate included EC958 (no cell death control) and the parent strain CFT073 TM (cell death control). The OD_600_ was recorded to ensure mutants had a similar growth pattern to that of the parent strain. 50 μL of overnight grown bacterial cultures were added to each well on the 96 well plate. Although 20 μL of overnight grown CFT073 TM was sufficient to completely kill HMDM, 50 μL was chosen in order to limit the identification of false positives. At 1 h post-infection, 200 μg/mL gentamicin (Life Technologies) was added to prevent extracellular bacterial growth.

### MTT assays

Supernatants were aspirated from the infected monolayer of cells on 96 well plates, after which cells were incubated with MTT reagent (1 mg/mL diluted in RPMI medium, Sigma) for 3–4 h at 37°C. Formazan crystals were dissolved in 100% isopropanol and the absorbance was read at 570 nm using a plate reader (PowerWave XS, Bio-Tek). A high MTT reading was indicative of viable cells, thus providing a primary screen for impaired killing of human macrophages by CFT073 TM Tn*5* mutants.

### LDH release assays

LDH release assays were used as a more direct read out of cell death to confirm CFT073 TM Tn*5* mutant phenotypes, and for subsequent assays of cell death. Cell culture supernatants were collected at the indicated time point post-infection, centrifuged at 500 g for 5 min and analyzed for LDH release using the In Vitro Toxicology Assay kit, as previously described [13].

### Western blotting

For each bacterial strain used, cultures were grown overnight, then resuspended at a cell density corresponding to OD_600_ of 1.0. Supernatants were precipitated overnight on ice using trichloroacetic acid (final concentration of 20%). Precipitated proteins were pelleted at 17,000 g for 30 min and washed twice with acetone, heat dried at 60°C for 30 min, rehydrated using 2 μL of 1 N sodium hydroxide, and resuspended in 100 uL of lysis buffer (125 mM Tris, 4% sodium dodecyl sulfate and 20% glycerol). Cell pellets were resuspended in the same lysis buffer as that of precipitated supernatants and then sonicated for 5 mins (Bioruptor, Diagenode). Western blotting was performed as previously described [64]. Membranes were treated with primary antibody against hemolysin (H10, mouse monoclonal antibody, isotype G1) [31], or outer membrane protein A (OmpA) (rabbit polyclonal antibody, Antibody Research Corporation) at a dilution of 1: 20,000 and 1: 10,000, respectively. Horseradish peroxidase-conjugated anti-mouse or anti-rabbit IgG antibodies (Cell Signalling Technology), which were used as secondary antibodies (1: 2,500 dilution), were detected using chemiluminescence and Super RX films (Fujifilm) or Amersham Imager 600 (GE health care limited).

For immunoblotting of HMDM cell lysates and supernatants, 4 × 10^5^ cells were used. Medium was replaced with fresh medium (OptiMEM, Invitrogen), after which cells were treated with or without LPS (100 ng/mL). At 1 h prior to infection, cells were treated with the NLRP3-specific inhibitor MCC950 or media alone. For analysis of secreted IL-1β, supernatants were precipitated with 4 volumes of acetone, then incubated overnight at -20°C. Samples were then centrifuged at 5,300 g/4°C for 30 min. Pellets were air dried and then dissolved in 50 uL lysis buffer. Cell lysates were prepared in the same volume of lysis buffer that were used to dissolve the pellet fraction. Western blotting was performed as previously described [64]. Membranes were probed with a primary antibody detecting both pro- and cleaved IL-1β (H-153, rabbit polyclonal Ig G, Santa Cruz) or Glyceraldehyde 3-phosphate dehydrogenase (GAPDH) (Trevigen) at a dilution 1:1,000 and 1:10,000, respectively. An anti-rabbit IgG antibody (Cell Signalling Technology) was used as the secondary antibody (1:2,500 dilution), and was detected using chemiluminescence and an Amersham Imager 600 (GE health care limited).

### Hemolytic assays

For comparisons between different bacterial strains, overnight cultures were resuspended at an OD_600_ of 0.1. Bacterial strains (10^6^ colony forming units) were then incubated with 5% defibrinated sheep blood and 10 mM calcium chloride at 37°C in a total volume of 1 mL, without shaking for 4 h. LB media alone and water were used as the blank and positive control, respectively. After 4 h incubation, samples were centrifuged at 6000 g for 2 min, and the OD of the supernatants were recorded at 540 nm. Hemolytic activity, calculated using the formula [(OD_sample_-OD_background_)/(OD_total_-OD_background_)]*100, was expressed as a percentage. EC958, which lacks the hemolysin genes, was used in parallel to quantitate any background signal associated with the assay. For each experiment, the values obtained with EC958 were subtracted from those of the CFT073 strains to correct for this background effect.

### qRT-PCR

Bacterial strains were grown at 37°C with shaking (200 rpm), and at exponential phase (an OD_600_ of 0.6), RNA was extracted using RNeasy QIAGEN and treated with Turbo DNase (Ambion, Invitrogen) to remove contaminating DNA. cDNA was synthesized from approximately 1 μg of RNA using SuperScript III reverse transcriptase (Invitrogen). cDNA generated was used in triplicates or quadruplicates for qRT-PCR using SYBR qPCR supermix (Invitrogen). Primers, used at a final concentration of 0.2 μM, were as follows: *hlyA*_Fwd: 5′-ggagcaggaggtttcagttgg-3′, *hlyA*_Rev: 5′-cgcgtggtcccaataagttc-3′, hlyC_Fwd: 5′-ggacttcaggtgatcgtaaatggt-3′, *hlyC*_Rev: 5′-ctgatggctcggaatagttcatc-3′, *gapA*_Fwd: 5′-ggccagcatatttgtcgaagttag-3′, *gapA*_Rev: 5′-ggtgcgaagaaagtggttatgac-3′. *gapA* was used as a housekeeping control gene. The reactions were performed using ViiA7 Real-time PCR system (Applied Biosystems), and relative transcript levels were normalized to *gapA* using the ΔΔCt method [65].

### ELISA

Cell culture supernatants were analyzed for IL-1β release by an ELISA kit (Duoset; R&D Systems), as per the manufacturer's instructions.

### Statistical analyses

Statistical significance was determined by either a t-test (two-tailed), or for multiple comparisons, a one-way ANOVA followed by Dunnett's test, using Prism7 software (GraphPad). A value of *p* < 0.05 was considered as statistically significant.

## Supplementary Material

Murthy_Supplementary_Revision2.docx
