# Supplementary material for: Regulation of hemolysin in uropathogenic Escherichia coli fine-tunes killing of human macrophages
Source: Virulence. 2018 May 30;9(1):967–80. doi: 10.1080/21505594.2018.1465786 (PMC5989160; doi:10.1080/21505594.2018.1465786)
Supplement: Murthy_Supplementary_Revision2.docx [file KVIR_A_1465786_SM8145.docx]

*
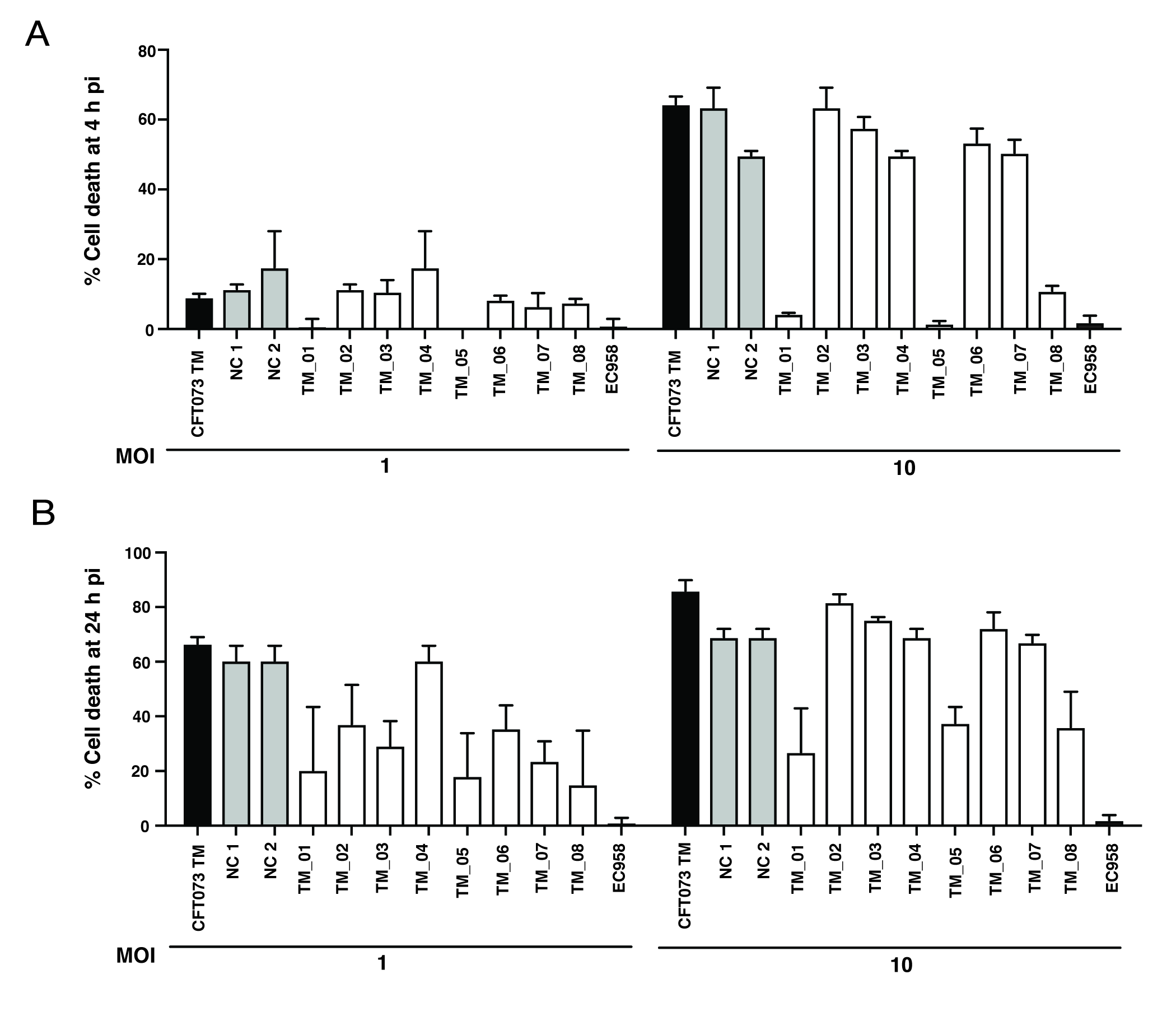
***Supplementary Figure 1: Validation of transposon mutants from the primary screen**. (**A**-**B**) HMDM were infected with the CFT073 TM parental strain (filled bars), two randomly-selected CFT073 TM Tn*5* mutants that behaved similar to the parental strain (NC 1, NC 2: grey bars), CFT073 TM mutants (hits from the Tn*5* screen, unfilled bars) or the EC958 control that does not cause cell death (MOI 1 and 10). Cell death was measured by LDH release assays at 4 h (**A**) and 24 h (**B**) pi. Data (mean+range, n=1) corresponds to a single experiment.


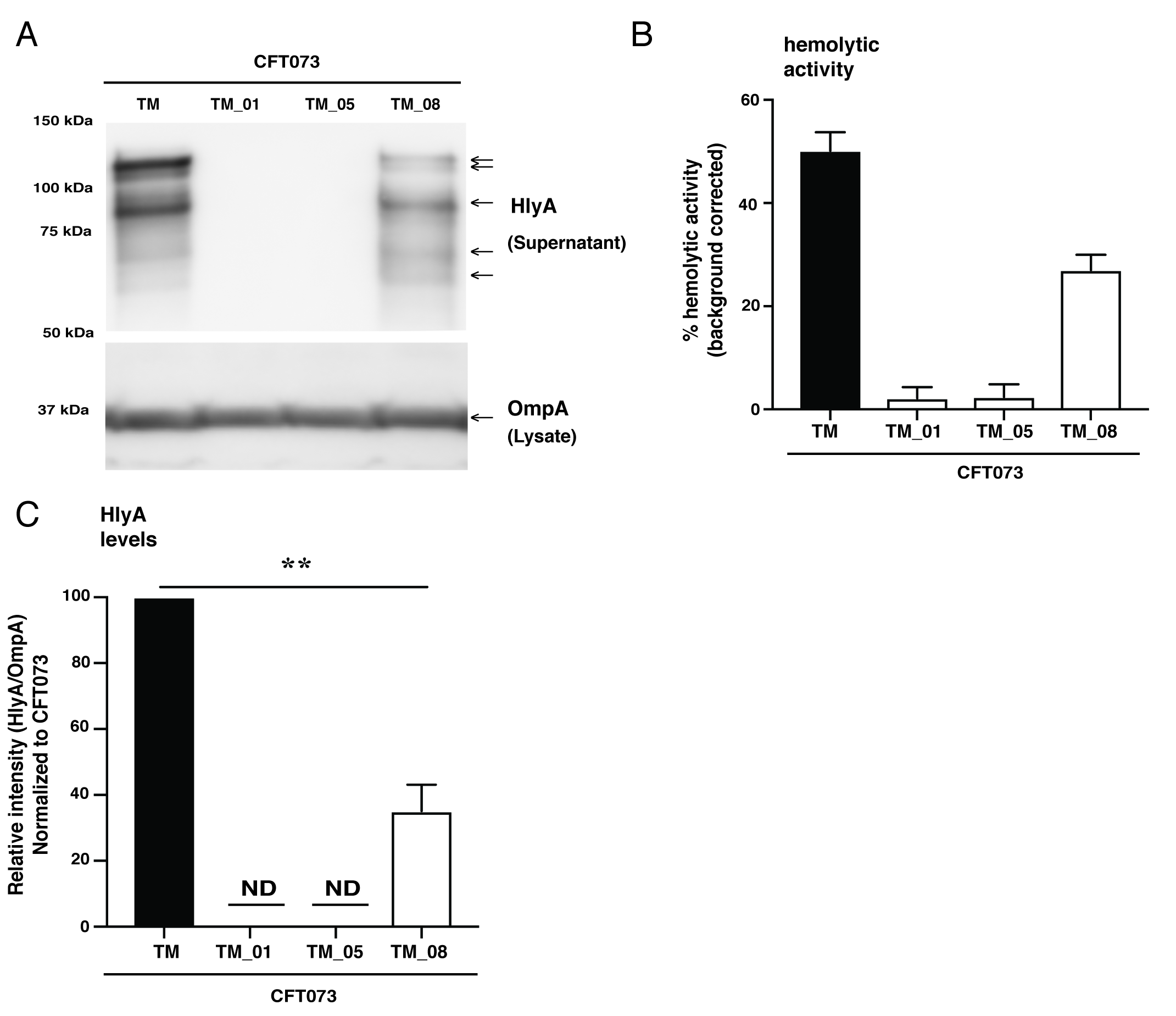


**Supplementary Figure 2: HlyA expression and activity of CFT073 TM and Tn*5* mutants.** (**A**) Strains were grown overnight in LB broth, and supernatants prepared from these cultures were concentrated and analyzed for HlyA expression by western blot analysis (arrows indicate full-length HlyA, as well as lower molecular weight HlyA bands). Corresponding cell lysates were prepared and OmpA levels were analyzed as a loading control. Data are representative of at least three independent experiments. (**B**) The indicated UPEC strains were grown overnight, then incubated with 5 % sheep red blood cells for 4 h, after which hemolytic activity was measured. Data (mean+range; n=2) are combined from two independent experiments. (**C**) Levels of HlyA, relative to OmpA, in immunoblots from (**A**) were quantified using Image J/Image analyzer (Amersham 600). For each experiment, HlyA levels in CFT073 TM were set as 100%. Data (mean+sem; n=3 to 5) are combined from five independent experiments (two of which did not include TM_01 or TM_05). ND denotes not detected. Statistical significance was determined by a two-tailed t-test (**p<0.01).

**Supplementary Table 1: Tn*5* insertions that reduced CFT073 TM-triggered killing of human macrophages.**

| **CFT073TM mutants** | **Locus tag** | **Gene** | **Product** |
| --- | --- | --- | --- |
| CFT073TM_01 | c3570 | hlyA | Hemolysin A |
| CFT073TM_02 | c4306 | yhiF | Transcriptional regulator (DNA binding motif) |
| CFT073TM_03 | c3625 | iucC | Aerobactin (iron uptake) |
| CFT073TM_04 | c4677 | rbsA | Ribosome import adenosine tri-phosphate binding protein |
| CFT073TM_05 | c3570 | hlyA | Hemolysin A |
| CFT073TM_06 | c4350 | yhjU | Hypothetical protein |
| CFT073TM_07 | c5204-c5205 | Intergenic region |  |
| CFT073TM_08 | c0562 | cof | Phosphatase belonging to HAD- like hydrolase |
